# Adera2.0: A Drug Repurposing Workflow for Neuroimmunological Investigations Using Neural Networks

**DOI:** 10.3390/molecules27196453

**Published:** 2022-09-30

**Authors:** Marzena Lazarczyk, Kamila Duda, Michel Edwar Mickael, Onurhan AK, Justyna Paszkiewicz, Agnieszka Kowalczyk, Jarosław Olav Horbańczuk, Mariusz Sacharczuk

**Affiliations:** 1 Department of Experimental Genomics, Institute of Genetics and Animal Biotechnology of the Polish Academy of Sciences, ul. Postepu 36A, Jastrzebiec, 05-552 Magdalenka, Poland; 2 Centre for Preclinical Research and Technology, Department of Pharmacodynamics, Faculty of Pharmacy with the Laboratory Medicine Division, Medical University of Warsaw, Banacha 1B, 02-091 Warsaw, Poland; 3 PM Research Center, Väpnaregatan 22, 58649 Linköping, Sweden; 4 Department of Sociology, Queen’s University at Kingston, 99 University Ave, Kingston, ON K7L 3N6, Canada; 5 Department of Health, John Paul II University of Applied Sciences in Biala Podlaska, Sidorska 95/97, 21-500 Biała Podlaska, Poland; 6 Institute of Genetics and Animal Biotechnology of the Polish Academy of Sciences, ul. Postepu 36A, Jastrzebiec, 05-552 Magdalenka, Poland; 7 Department of Pharmacodynamics, Faculty of Pharmacy with the Laboratory Medicine Division, Medical University of Warsaw, Banacha 1B, 02-091 Warsaw, Poland

**Keywords:** drug repurposing, neuro-immunology, deep neural network

## Abstract

Drug repurposing in the context of neuroimmunological (NI) investigations is still in its primary stages. Drug repurposing is an important method that bypasses lengthy drug discovery procedures and focuses on discovering new usages for known medications. Neuroimmunological diseases, such as Alzheimer’s, Parkinson’s, multiple sclerosis, and depression, include various pathologies that result from the interaction between the central nervous system and the immune system. However, the repurposing of NI medications is hindered by the vast amount of information that needs mining. We previously presented Adera1.0, which was capable of text mining PubMed for answering query-based questions. However, Adera1.0 was not able to automatically identify chemical compounds within relevant sentences. To challenge the need for repurposing known medications for neuroimmunological diseases, we built a deep neural network named Adera2.0 to perform drug repurposing. The workflow uses three deep learning networks. The first network is an encoder and its main task is to embed text into matrices. The second network uses a mean squared error (MSE) loss function to predict answers in the form of embedded matrices. The third network, which constitutes the main novelty in our updated workflow, also uses a MSE loss function. Its main usage is to extract compound names from relevant sentences resulting from the previous network. To optimize the network function, we compared eight different designs. We found that a deep neural network consisting of an RNN neural network and a leaky ReLU could achieve 0.0001 loss and 67% sensitivity. Additionally, we validated Adera2.0’s ability to predict NI drug usage against the DRUG Repurposing Hub database. These results establish the ability of Adera2.0 to repurpose drug candidates that can shorten the development of the drug cycle. The workflow could be download online.

## 1. Introduction

Drug repurposing represents a lifeline for the neuroimmunological drug industry. The rate of development of new drugs has dramatically slowed in recent years [1]. This phenomenon was accompanied by a significant increase in the cost of drug development [1]. A single novel drug might cost 1 billion USD to produce [2]. Drug repurposing constitutes a viable alternative to conventional drug development techniques. Repurposing drugs that have already been legalized for human usage can substantially reduce the costs accompanying the primary stages of drug discovery [3]. Moreover, repurposing novel drugs eliminates the delay faced by de novo drug development [4]. At present, numerous repurposed medications are being extensively used, such as azathioprine, which is commonly used for rheumatoid arthritis; currently, it is used for renal transplants [5]. Minoxidil, originally intended to treat ulcers, was repurposed for the treatment of severe hypertension when early studies revealed that it was a vasodilator [6]. Another example is thalidomide, which was initially licensed in the 1950s to treat morning sickness in expectant mothers before being repurposed in 2006 to treat multiple myeloma [7]. In addition, although chlorpromazine was initially tested as an antimalarial medication, it was shown to be more effective in the treatment of mania [8]. We previously repurposed zileuton, which was originally used as an inhibitor of the 5-lipoxygenase, as an Nrf2 activator to treat depression [4]. Thus, drug repurposing techniques have proven essential for improving the drug production cycle.

Several computational approaches aim to extract drug information from medical literature using question–answer drug repurposing systems. These approaches could be applied separately or as a part of a workflow. These approaches include data mining, network analysis, and machine learning [9]. (I) The data mining approach is based on text mining. Text mining is utilized to identify drug names, using part-of-speech tagging or the bag of words algorithms. Text mining approaches include polysearch [10], DNorm [11], and MEDIC [12,13]. The main drawback of this approach is the need for a large-sized dictionary to predict textual relationships. The dictionary used requires various resources to perform several processing steps, such as normalization, mapping, and maintenance. An alternative approach is to directly text mine databases (e.g., DrugBank) [14]. However, many of the chemical compounds currently investigated are not yet accessible through DrugBank. Another text mining approach is based on the use of semantics technologies. Semantic text mining substitutes the need for stored vocabulary with a semantic concept built of multi-word terms (ngrams) [15]. This system could prove useful in filtering out less relevant documents. However, it suffers from the need to store the semantics database. An alternative method of text mining is based on ABC occurrence (i.e., if A and B occur together, B and C occur together, then A and C may occur together) [16]. However, this system suffers from low sensitivity (a high degree of false positives). (II) Networks analysis techniques are being widely employed in drug repurposing approaches. These approaches do not only detect drug compounds but can also mine drug characteristics, such as usages, indications, actions, and targets [12]. A bipartite graph is a relevant example that demonstrates this approach. Hu and Agarwal combined the analysis of microarray expression profiles with CMap data to produce a network of drugs–genes [17]. The drawback of this workflow is that future investigations are limited to the microarray datasets investigated. Clustering techniques based on the calculated Jaccard coefficient for Kegg Medicus have also been utilized [18]. The disadvantage of this system is that it is limited by the Kegg database annotation [19] (III) Machine learning techniques utilized to extract drug information from immunological texts could be generically grouped into classification or sequence labeling while using regression-based approaches is less common [12]. Classification models employed to recognize drug entities include maximum entropy (ME) and support vector machine (SVM) [20,21]. One of the drawbacks of these classification methods is that they are unaware of the order of the tokens (e.g., words). 

Combining natural language processing (NLP) techniques with machine learning could be used to increase the awareness of the order and relationships between entities in any given text [22]. The primary objective of NLP techniques is to recognize concepts and relationships. A typical NLP workflow includes text tokenization to detect words and sentence boundaries. Part-of-speech algorithms could be used to tag the type of words using different tags (e.g., nouns, verbs, and prepositions). Extracted words are then mapped into a biological category. Finally, a tree is built based on the text syntax [23]. Additional NLP functionality includes several methods, such as (i) assertion status detection, which classifies a medical term according to its status into one of four groups, namely “present”, “absent”, “conditional”, or “associated with someone else” [24]. Assertion status detection methods have been combined with AI methods, such as convolutional neural networks (CNNs) and long-short-term memory networks (LSTMs) [23]. (ii) Entity resolution is an intriguing concept that attempts to comprehend the relationships between words within a sentence (e.g., links possessive articles to the correct nouns) [25]. This method is used with LTSM and achieves high accuracy. However, it does not extract compound names. (iii) Relationship extraction techniques aim to extract semantic associations between two or more entities (e.g., drug–disease relationships) [26]. (iv) Named entity recognition (NER) techniques implemented using the hidden Markov model (HMM) or conditional random field(s) (CRF) are aware of the sentence order and have been shown to outperform classification methods on a variety of tasks [27]. (v) Recently, word-representation features seemed to show the highest efficiency [28]. Word representation features are generated through unsupervised machine learning algorithms on unstructured texts. One of the advantages of using word representation is that this method takes into account the semantic information of the words. Currently, Word2VEC is one of the most widely used methods [29]. However, embedding for the whole sentence has achieved higher performance. In the case of query–answer scenarios, the distance between the embedded matrices of the question and the answers is used as an indication of the relevance between the question and the answer. However, the performances of sentence embedding-based systems in this context are limited [4].

In the current study, we developed a question–answer system based on AI, we call it Adera2.0. The input to the system is a user query (i.e., a question). The system is composed of several phases. In the first phase, the PubMed IDs relevant to the question posed by the user are retrieved from PubMed using a Python function. The downloaded PDFs are then parsed using Tika and stored in JSON format [30]. In the next phase, we utilized the universal autoencoder to embed each sentence in each parsed PDF into a 1 × 512 matrix. Following that, we use our relevance network to classify sentences within each PDF based on their relevance to the given question [4]. This network input is the 512 × 1 matrix embedding of the posed question. The output is a matrix embedding of a predicted answer based on an optimized loss regression function. Embedding matrices are sorted based on their distance from the predicted answer matrix. In the next phase, we built an extraction network. Its primary function is to extract drug names from relevant sentences identified in the previous phase. To achieve this output, each sentence is parsed into words and embedded using the universal encoder. After that, we used the extraction network to predict a generic compound embedding. Parsed words were sorted based on their distance from the predicted embedded matrix. We compared the performance of eight network designs to achieve the task of extracting compound names. We cross-validated Adera2.0’s performance against a gold standard dataset (Drug Repurposing Hub, Broad Institute dataset) and the current literature [31]. We investigated repurposing anti-oxidant compounds that could be used to regulate Th17 cells in depression. Previously, we found that Th17 infiltrates the blood–brain barrier through a paracellular route causing depression-like behavior [32,33]. The main objective of the in silico validation step was to find anti-oxidant drugs that could be used to reduce the effect of Th17 in depression and score at least four out of five on Lipinski’s score. Out of the top ten compounds generated by our software, two were shown to be true candidates. 

## 2. Methods

### 2.1. Overview of the Workflow

The workflow consists of four main phases: (i) building a JSON database, (ii) sentence embedding, (iii) computation of relevance, and (iv) extraction of compound names. 

**Figure 1:**
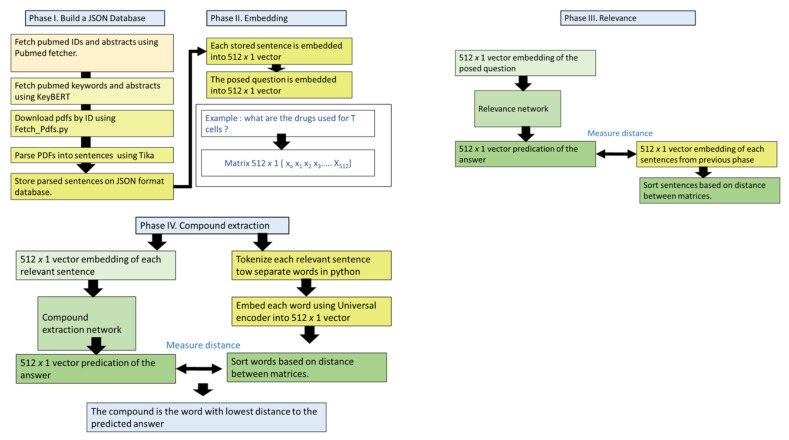
A detailed workflow of our proposal. There are four phases in the workflow. The user first enters a disease or a primary pathway to be investigated. The search step is done to select relevant PubMed articles. Then each accepted article is sorted and parsed. The first network converts each sentence in each PDF to a single embedding matrix. The second neural network sorts each sentence in each PDF based on its relevance to the query. The third neural network extracts drug names from each relevant sentence.

The first phase of the workflow (phase I) covers the aim of building a database of the JSON format containing parsed PDFs (Figure 1). This phase consists of five steps. The first step’s objective is to fetch the PubMed IDs related to the search query. This is accomplished by using the PubMed fetcher function available through the Metapub python library. This step uses the input query to search for recent PubMed articles that match the query terms. After that, in the second step, the workflow fetches the abstracts and keywords of the retrieved PubMed IDs. This is achieved through the use of the python library Keybert. The third step in this phase involves downloading the identified PDFs; this is done using the fetch_PDFs library. The fourth step of this phase is the parsing of each PDF into separate sentences and storing all PDFs in a single database using the TIKA library [30]. The fifth step includes writing all parsed sentences into a single JSON format. 

The second phase constitutes sentence embedding. This is done by employing the universal encoder. The main function of this network is to embed each parsed sentence in the JSON dataset into a 512 × 1 matrix. The universal encoder uses a deep averaging network as its main architecture. In this system, the word embedding is averaged. The output of this step is used as the input for a feedforward deep neural network to generate sentence embedding. The training data for this model include (i) unsupervised training datasets from Wikipedia, web news, and question and answer websites. (ii) Additionally, the neural network was trained on the supervised data of the Stanford natural language inference system [34]. It is important to note that the Pearson correlation was used to estimate the model’s accuracy. The model was used to predict the similarity between sentence pairs. The test was validated against human judgment. Overall, the model accuracy was estimated to be 0.76.

The objective of the third phase is to measure the relevance between the embedding of the parsed query (i.e., question) and the embedding of each parsed sentence in the JSON database. This phase is performed through a neural network. The input of the neural network is the embedding matrix of the query. The network uses a MSE loss function with an adaptive moment estimation (ADAM) optimizer. The network was trained on the entries sampled from the SciQ dataset. SciQ is a scientific answer dataset of 13,679 entries divided into 3 sub-datasets (training, test, and validation) [35]. The output of this network is a 512 × 1 matrix that represents the prediction of the answer embedding. Then, all the sentences in the JSON database are sorted based on the square root of the distance between the embedding matrix of each of them and the output of the second neural network (i.e., the predicted answer).

The fourth phase’s purpose is to extract the compound names from each answer resulting from the previous step. To achieve that, we designed a deep neural network. The inputs of this network are the sorted embedding matrices resulting from phase 3. The network was trained on an in-house-generated database [36]. The database is a collection of one thousand manually curated entries. The database consists of two columns representing the sentence–drug relationship. The first column includes sentences retrieved from; PubMed articles, Drug bank [14], and the n2c2 NLP Research data sets [37]. The second column represents the drug compounds, which were manually identified in each sentence. The dataset was divided into three smaller-size datasets as follows: (i) training dataset with 500 entries, (ii) test dataset with 250 entries, and (iii) validation dataset with 250 entries. It is important to note that our current trained network can be used for transfer learning when new categories of data or a larger set of validated drug entries are available. Similar to the relevance network, the extraction network uses an MSE loss function with an ADAM optimizer. We compared the performance of eight different architectures for this network (Figure 1, Table 1 and Table 2). Given an embedded sequence in 512 × 1 matrix format, the network predicts a word embedding in a 512 × 1 matrix format. Then the distance between the embedding of each word of each of the identified sentences and the predicted embedding matrix is calculated. The distance between the matrices is considered to correlate linearly to the similarity between the predicted answer and each embedding word. To measure the performance of our extraction network (E), we performed a sensitivity analysis (Equation (1)). Additionally, we compared the sensitivity values with random sampling.

(1) $$\mathrm{sensitivity}=\frac{\mathrm{Number}\;\mathrm{of}\;\mathrm{True}\;\mathrm{predictions}\;}{\mathrm{Number}\;\mathrm{of}\;\mathrm{True}\;\mathrm{predictions}+\mathrm{Number}\;\mathrm{of}\;\mathrm{False}\;\mathrm{predictions}},$$

### 2.2. Cross Validation Using Known Drugs

We validated Adera2.0’s abilities by measuring its sensitivity. We compared the performances of our eight novel networks, focusing on their ability to extract drug names from sentences (phase 4). We utilized three strategies: (i) posing a question based on a disease. (ii) posing a question based on a specific type of drug, and (iii) posing a question pertaining to a specific pathway. Sensitivity (Equation (1)) was estimated for each model. After that K-means clustering was used to compare the sensitivity levels between various models investigated.

### 2.3. Case Study

The objective of the case study is to repurpose a compound that could target the inflammatory process regulated by Th17 infiltration of the brain during depression [32]. The constraints on the characteristics of the repurposed drug include its ability to inhibit Th17 differentiation, enhance depression prognosis and pass through the blood–brain barrier as well as being biologically safe [38]. The question used to query the system is “What are anti-oxidant drugs”. The output from our model was further filtered by examining the drug’s physicochemical properties. The number of violations of Lipinski’s rule was calculated using molinspiration server (www.molinspiration.com) accessed on 15 August 2022 [39]. The clogP, solubility, mol-weight, Tpsa, drug-likeness, drug toxicity, mutagenicity probability, tumorigenicity, irritant-ability, and reproductive effectiveness drug score, were determined by employing Osiris Property Explorer (OPE; http://www.organic-chemistry.org/prog/peo/) accessed on 15 August 2022. We utilized the BBB predictor (http://www.cbligand.org/BBB), accessed on 15 August 2022 to predict the ability of all the repurposed compounds to cross the blood–brain barrier. 

### 2.4. Availability and Implementation

To facilitate the use of Adera2.0, we have developed a Windows©-compatible workflow that is quick and reliable. The application with user instruction pages could be found on the GitHub repository at https://github.com/michel-phylo/ADERA2.0.

## 3. Results 

### 3.1. Workflow 

**Figure 2:**
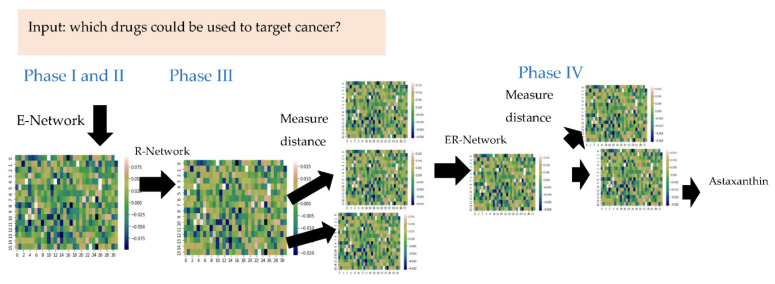
Workflow description. Phase (I&II); After parsing PDFs into single sentences, each sentence is embedded into A 512 × 1 vector (here reshaped into 32 × 16) using the embedding (E) network. Phase (III); the relevance (R) network predicts the answer to the embedded question. The form of the predicted answer is a 512 vector. The distance tween the predicted answer and the embedding of each sentence is calculated. The most relevant sentences are estimated to possess the shortest distance from the embedded question matrix. After that, in phase (IV), the extraction (ER) network predicts the compound embedding for each relevant sentence. The distance between each word embedding and the predicted answer embedding is calculated. The word with the shortest distance between its matrix embedding and the predicted compound embedding is estimated to represent the compound name.

Adera2.0 is a python-based neural network workflow specially designed to repurpose drugs pertaining to the field of immunology. Adera2.0 consists of three neural networks. Its main novelty is its third neural network structure. The first network is a universal autoencoder. The second structure is a relevance network based on a similar design used in our previous publication called Adera1.0 [4]. To that end, Adera2.0 receives a user query, performs a PubMed search, embeds the query, parses, and embeds each sentence in the retrieved PubMed PDFs. Following that, the distances between the matrices are calculated. The matrix with the shortest distance to the predicted embedding matrix is considered to represent the most relevant sentence. The novel-added network’s primary function is extracting drug compounds from relevant sentences resulting from the previous network (Figure 2). This is done by feeding the network the embedding matrix of the query. The network predicts an embedding representing a generic compound. After that, the distance between each word embedding and the predicted matrix is calculated. The word with the shortest distance between its matrix embedding and the predicted compound matrices is hypothesized to represent the compound.

### 3.2. Results of the Novel Neural Network

**Figure 3:**
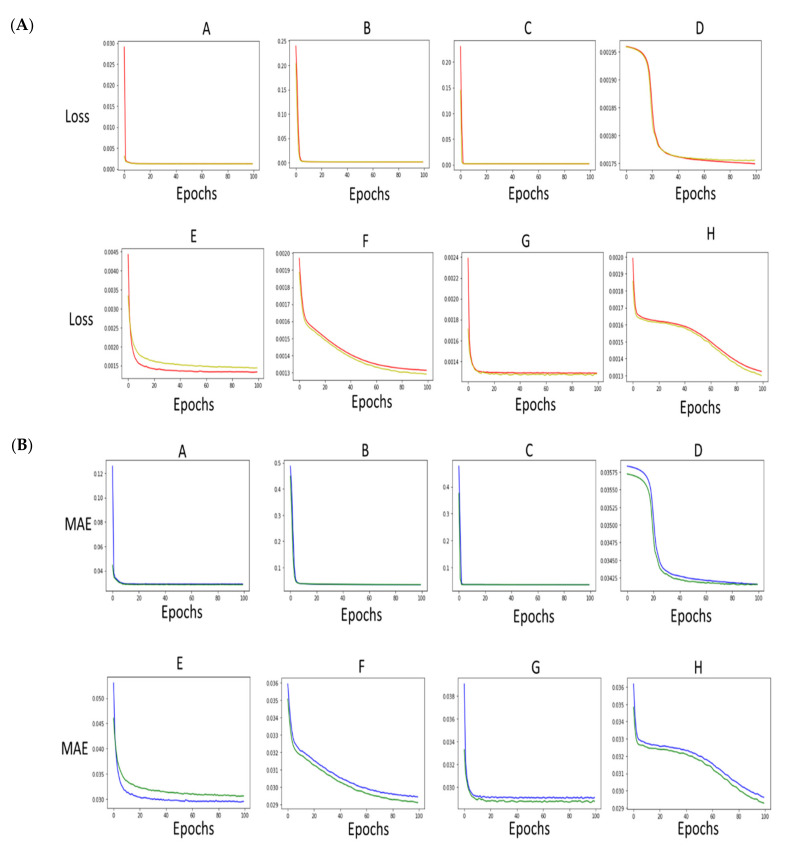
Loss and mean absolute error functions of the extraction network in Adera2.0 workflow. (**A**) Models A, E, F, G, and H achieve the lowest loss. Models B, C, D and H show the highest loss. Green is utilized for the loss function, while red is used for the validation loss calculations. (**B**) Model A achieves the lowest MAE value. The mean absolute error is in blue, while the validation of the MAE curve is in green. It is worth noting that the MAE validation values are lower than their respective MAE training values. The MAE is calculated over any given dataset. The dataset is split randomly into training and validation datasets with a ratio of 0.61:0.39. Thus the size of the validation dataset is smaller, resulting in slightly lower MAE values compared to the MAE values resulting from the training dataset.

**Figure 4:**
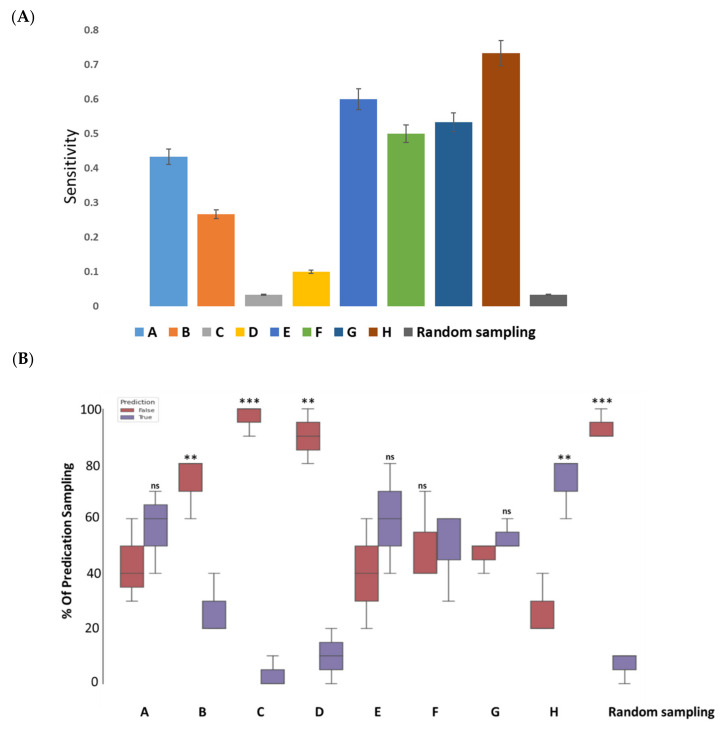
Sensitivity analysis for Adera2.0 extraction networks. (**A**) Normalized sensitivity for different architectures of the extraction network (**B**) Boxplot of True and negative prediction for various architectures of the extraction network. ** represents *p*-value of <0.001, *** represents *p*-value of <0.0001, while ns stand for non-significant.

The third neural network extracts compound names from sentences. The design of the network layers played a vital role in determining the loss value. We compared the performance of eight different designs based on their ability to extract compound names from sentences (Table 1 and Table 2, Figure 3 and Figure 4). The core of networks A, EF, G, and H is an RNN network, while designs B, C, and D only possess dense layers with various activation functions (Table 1 and Figure 3). Networks A, E, F, G, and H achieved the lowest loss of 0.0013 and the lowest mean absolute error (0.02). Similar results were achieved on the test and the validation datasets. Conversely, networks B, C, and D achieved the highest loss (0.0017, 0.0019, 0.0017 respectively). Their poor performance was mirrored in the high mean absolute error on the validation network of 0.0377, 0.0348, and 0.0339 respectively (Table 2). It is noticeable that networks A, B, C, and G reached their maximum performance with lower than 20 epochs. The difference between training and validation network D seems to be increasing after 50 epochs, suggesting this particular network did not converge. Network F, H continued to learn even after more than 90 epochs, indicating that they perform better than the rest of the designs.

**Table 1:** Different structures used for network number (3): Extraction network.

Architecture	Description
A	Dense(512, activation = ‘selu’) Dense(512, activation = ‘sigmoid’) Dense(512, activation = ‘relu’) Dense(512, activation = ‘relu’) Dense(512, activation = ‘selu’) LeakyReLU() tf.keras.layers.Activation(‘selu’) SimpleRNN(512) Dense(512, activation = ‘selu’) Dense(512, activation = ‘relu’) Dense(512, activation = ‘selu’) Dense(512, activation = ‘relu’) Dense(512, activation = ‘selu’)
B	Dense(512, activation = ‘selu’) Dense(512, activation = ‘sigmoid’)
C	Dense(512, activation = ‘relu’) Dense(512, activation = ‘selu’) Dense(512, activation = ‘sigmoid’)
D	Dense(512, activation = ‘selu’) Dense(512, activation = ‘Softmax’) PReLU()
E	Dense(512, activation = ‘selu’) SimpleRNN(512)
F	Activation(‘selu’) RNN(tf.keras.layers.LSTMCell(512)) LeakyReLU() LeakyReLU()
G	Dense(512, activation = ‘selu’) PReLU() Dense(512, activation = ‘selu’) SimpleRNN(512) Activation(‘selu’) LeakyReLU()
H	Activation(‘selu’) RNN(tf.keras.layers.LSTMCell(512)) PReLU() LeakyReLU() LeakyReLU()

**Table 2:** Loss and MAE comparison for Extraction network various architectures.

	Train Dataset				Test Dataset		Validate	
Architecture	Loss	Mean Absolute_Error	Validation Loss	Validation Mean Absolute Error	Loss	Mean Absolute_Error	Loss	Mean Absolute_Error
A	0.0013	0.0290	0.0013	0.0286	0.0013	0.0288	0.0012	0.0283
B	0.0017	0.0333	0.0018	0.0338	0.0020	0.0354	0.0025	0.0377
C	0.0019	0.0351	0.0018	0.0349	0.0018	0.0350	0.0018	0.0348
D	0.0017	0.0342	0.0018	0.0342	0.0018	0.0341	0.0017	0.0339
E	0.0013	0.0295	0.0014	0.0306	0.0015	0.0309	0.0015	0.0310
F	0.0013	0.0294	0.0013	0.0291	0.0013	0.0293	0.0013	0.0290
G	0.0013	0.0291	0.0013	0.0287	0.0013	0.0293	0.0013	0.0290
H	0.0013	0.0296	0.0013	0.0293	0.0013	0.0294	0.0013	0.0292

### 3.3. Sensitivity Analysis 

We performed a sensitivity analysis to examine the performance of our proposed extraction networks. Our results indicate that the highest achieving architecture is network H, as it achieved a mean of 73.2% ± 11.5 with a *p*-value of 0.007 (Figure 4). In the case of a random sampling of data extraction, the sensitivity falls to 6.9 ± 3 % (*p*-value < 0.0001). Overall, our data indicate that compound extraction model H significantly outperforms random sampling of the data (*p*-value < 0.0007). Using the profiler function in TensorFlow we analyzed the relative time and memory efficiency values for the models used. We used the GoogleColab Nvidia K80/T4 (host machine) and NVIDIA GeForce GT 705 (local machine) to execute the training, testing, and validation process. Our results indicate that using RNN layers increases computation time, with networks AEFGH having a mean of 7.04 ± 1.87 ms, while networks BCD have a mean was 4.57 ± 0.20 ms. It could be noted that having multiple layers, as in the case of model A, resulted in having a slightly higher peak memory usage using the local device and on the Google Cloud host (Supplementary Materials).

### 3.4. In Silico Validation of Adera2.0 Ability to Mine Known Drugs

**Figure 5:**
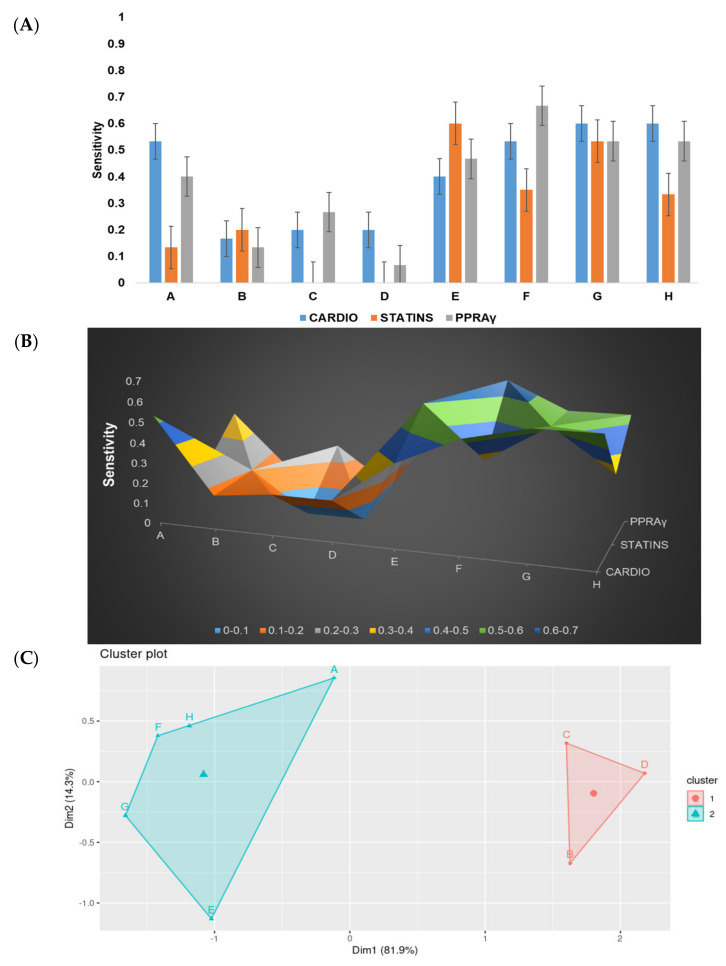
Sensitivity analysis of the investigated models based on their ability to identify known drugs. To validate the performance of our models, we compared their performance using three types of questions. (**A**,**B**) We found that models G and H achieve the highest sensitivity on generic questions. (**A**,**B**) Model F could be more suitable for the pathway question–answer category. (**C**) Our K-means clustering analysis indicates that the models separate based on their sensitivity into a cluster containing the B, C, and D models and another cluster containing the models’; A, E, F, H, and G.

We validated Adera2.0’s abilities by measuring its sensitivity to extract known drug names. We compared the performances of our eight novel networks, focusing on their ability to extract drug names from sentences (Phase IV) (Figure 5A–C). To measure the sensitivity, we used three groups of sentences (each group consisting of five sentences). These groups were formed based on their types into (i) generic, (ii) specific to a drug family, and (iii) specific to a drug pathway. For the first category, we used the question “what are the drugs commonly used to treat cardiovascular diseases?”. For this category, Models G and H had the highest mean of 0.6 with a standard deviation of 0.15 (ii) In the case of the second category, we used the question; “What are the types of statins?”. All the models scored lower accuracy, with the highest being Model E (scoring 0.6). (iii) In the third category, we used the question, “Which drugs function as PPARγ agonists?”. In this category, Model F scored the highest with a value of 0.67. Overall, Model F was more accurate in extracting drug names from sentences related to biological pathways. Models G and H seemed to be more suitable for sentences acquired from more generic inquiries.

### 3.5. In Silico Validation Demonstrates Adera Performance

Adera2.0 is the new version of our previous publication (Adera1.0) [4]. The main differences between the two network architectures are highlighted in Table 3. In summary, we updated our workflow of Adera1.0 considerably to include multiple novel features. We added a neural network capable of extracting compound names from sentences. Network “H” was chosen to build the extraction network after comparing eight different neural network architectures. The neural network accepts the embedding matrix of the user query and outputs a 512x 1 matrix. The distance between the output matrix and the word embedding matrices of each relevant sentence is calculated to determine the shortest distance. Using this approach; a single compound name is extracted from each relevant sentence. 

We demonstrated the ability of our workflow to repurpose drugs, by showing its ability to repurpose compounds based on their abilities to regulate the Th17 function in depression diseases. A query question was used to initiate the workflow. The query question was given as: “what are anti-oxidant drugs?”. The workflow produced ten compounds (Table 4). It could be noticed that the resulting compounds belong to different chemical and physical families. For example, coumarin is a phenylpropanoid used by plants to fend off animals [40]. It is mainly used as an anticoagulant. On the other hand, glycyrrhetinic acid is a triterpenoid derived from the root of licorice and could function as an anti-cancer and anti-inflammatory drug [41].

**Figure 6:**
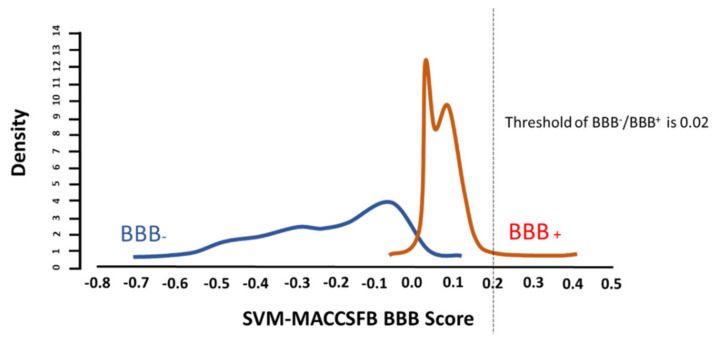
BBB infiltration predictions of bicalutamide.

To examine the feasibility of our predictions to be used as Th17 inhibitors in depression, we subjected the compounds to 11 constraints. In the first set of constraints, pathogenicity was measured (Table 5). Several compounds were predicted not to have any pathogenicity, such as acebutolol, glycyrrhetinic acid, glycyrrhizin, porphyrin, bicalutamide, and fucoxanthin. Conversely, amygdalin, on the other hand, is predicted to be of high risk in three of the four categories, highlighting the limitations of our workflow in terms of being inherently unaware of toxicity. Moreover, our investigation of the physical and chemical properties of the repurposed compounds revealed that various compounds did not break any of Lipinski’s rules, such as acebutolol, beta-D-fructofuranose, coumarin, resveratrol, and bicalutamide, while glycyrrhizin and flavonoids broke three rules, deeming them weak on the level of oral absorbance. Furthermore, as our repurposing tasks are related to the ability of the compound to inhibit pathogenic Th17 function in the brain during depression, one of the main characteristics of the required drugs is blood–brain barrier infiltration ability. Our investigation revealed that coumarin, resveratrol, porphyrin, and bicalutamide were able to cross the BBB (Table 6 and Figure 6). We validated our findings by searching relevant literature and the Drug Repurposing Hub (Table 7). Notably, bicalutamide was inferred to decrease Th17 activity by inhibiting NF-κB signaling [42]. Paradoxically, it was reported to worsen depression prognosis. Conversely, the porphyrin derivative known as hematoporphyrin is regularly used as an antidepressant and antipsychotic. It was also used to inhibit the podoplanin–CLEC-2 interaction and decrease the incidence of metastasis and thrombosis [43]. Conversely, another derivative of porphyrin known as tin protoporphyrin IX was shown to inhibit Heme oxygenase-1, which may affect Th17 activity [44]. 

Bicalutamide scored 0.02 on the SVM-MACCCSFP BBB score indicating that it is highly likely to be able to cross the BBB.

## 4. Discussion 

Our system offers a drug repurposing workflow. Thousands of literature documents are being added to PubMed and similar repositories each year. Mining drug databases is a viable option for drug repurposing. However, classical approaches use large dictionaries to extract the compound from mined sentences. Word2VEC approaches offer a solution by embedding text to numerical matrices. However, it lacks awareness of sentence context. Our updated workflow is both able to determine sentence relevance and also to extract compound names with a loss of less than 0.01 and a sensitivity of 67% (Table 2, Figure 4 and Figure 5).

### 4.1. Output of the Extraction Network Highlight the Importance of Architecture 

Our approach, as well as others, demonstrates that neural network prediction results are highly dependable on the architecture and the loss function. The extraction network’s main task is, given a sentence embedding, to predict a word embedding representing a generic compound. To do this, we trained our network on an in-house dataset of 1000 entries. Our findings, in agreement with previous reports, suggest that RNN could be most suited for compound name extraction tasks. Notably, designs that lack the RNN structures perform significantly worse (Figure 3 and Figure 4). This observation was further validated by inspecting the ability of our eight models to extract known drug names. K-means clustering analysis (Figure 5C) showed clear clustering between Models A, E, F, G, and H (cluster I) and models B, C, and D (cluster II). RNNs are known to perform better than CNN-based models using a multi-classifier framework. CNN was reported not to be able to grasp the text context [60]. RNNs can predict time series and have been used successfully for text normalization, de-identification, and sequence labeling [61,62]. However, RNNs also suffer from low efficiency in multi-classifier settings [63]. In this report, we proposed a novel network design to extract drug compounds from sentences using a regression loss function (i.e., mean square root error (MSE)). Our model “H” which utilizes a simple RNN achieves a loss of less than 0.0013 as well as a sensitivity of an average of 67% (Figure 3, Figure 4 and Figure 5). 

### 4.2. Case Study Results Show Our Workflow Predictions Accuracy 

We demonstrated the ability of our workflow to correctly repurpose a compound that can target Th17 expansion in the brain. We have previously shown that Th17 is capable of infiltrating the brain during depression diseases, causing a localized inflammation of the hypothalamus. This inflammation is associated with depression-like behavior [64,65,66]. Currently, depression drugs focus on the role of neurotransmitters and overlook the inflammatory side [67]. Thus, repurposing drugs to target the inflammatory symptoms of depression can help ease the symptoms and ensure a better quality of life. We used Adera to mine PubMed PDFs for anti-oxidant drugs. We investigated a list of ten drugs for their ability to fulfill the needed objective (i.e., target Th17 expansion in the brain during depression). Additionally, we subjected the resulting drugs to a rigorous list of constraints that ensured that the repurposed drug would fulfill the objective while being biologically safe, orally available, and non-toxic. Our investigation proposes the use of porphyrin and bicalutamide. Our findings are supported by the literature, which shows that both drugs have interacted with the effect on Th17 and are regularly used to improve the prognosis of depression [31,38,44].

### 4.3. Limitations and Future Direction

Currently, Adera2.0 is unaware of the structure of the repurposed drug. Downstream analysis using molecular dynamics, molecular docking, and QM/MM programs are still essential to reduce the cost of the in vitro and in vivo validation of drugs that could be repurposed using our software. Future improvements to the software will include neural networks predicting protein–drug interactions to overcome this limitation.

## 5. Conclusions

Our workflow (Adera2.0) is capable of text mining highly specific PDFs to search for drugs that could be used in an immunological context. Our workflow is capable of automatically downloading and parsing PDFs from PubMed. The workflow predicts the relevance between each parsed sentence and the user query. The workflow extracts compound names from relevant sentences with a sensitivity in the 60–70% range. Overall, the use of Adera may reduce the time and costs required for R&D in drug discovery.

## Figures and Tables

**Table 3:** Comparison between Adera1.0 and Adera2.0.

Description	Adera1.0	Adera2.0
Function	Question–answer system (QA) to find the nearest answer to a given query	
Input to the system	User input (query question)	
Overall number of networks	2	3
Networks used	Embedding and relevance	Embedding, relevance, and extraction
Embedding network function	Generate embedding for query question and each sentence in the relevant PDFs	
Embedding network architecture	DAN (deep averaging network) followed by feedforward autoencoder	
Training of the network	Wikipedia, web news, and question and answer websites	
Pearson correlation performance	0.76	
Output of the embedding network	512 × 1 embedding matrix	
Relevance network function	Determine the relevance of the answers	
Relevance network architecture	Convolution network	
Training of the network	Small in-house dataset	SciQ
Best loss function performance	0.0018–0.002	
Output of the relevant network	Embedding matrices sorted based on relevance to the answer	
Extraction of compounds from sentences function	Functionality does not exist	To extract compound names from the relevant sentence
Relevance network architecture		eight different architectures
Best loss function performance		0.0013
Input		Sorted sentences
Training of the network		Dataset Extraction (1.0)
Output		List of compounds

**Table 4:** Summary of the compounds identified using our software.

Compound	Molecule Structure	Biological Activity
Acebutolol	(O=C(N)Cc1ccc(cc1)OCC(O)CNC(C)C)	Acebutolol is a selective 1-receptor antagonist that lowers blood pressure and heart rate.
beta-D-fructofuranose	C(C1C(C(C(O1)(CO)O)O)O)O	beta-D-fructofuranose plays a key role in glycolysis. However, its exact function is still unknown. Currently, it is in a clinical trial.
		(clinicaltrials.gov Identifier: NCT05207488)
Elatin (flavonoid)	CC1C(C(C(C(O1)C2=C3C(=C(C(=C2O)C4C(C(C(C(O4)CO)O)O)O)O)C(=O)C=C(O3)C5=CC(=C(C=C5)O)O)O)O)O	Elatin was reported to be antioxidant, anti-cancer, anti-microbial, neuroprotective, and anti-inflammation.
Coumarin	O=C1C=Cc2ccccc2O1	Coumarin is an anticoagulant used to treat deep vein thrombosis and pulmonary embolism.
Resveratrol	Oc1ccc(cc1)C=Cc1cc(O)cc(c1)O	Resveratrol was reported to reduce markers of inflammation.
Glycyrrhetinic acid	CC1(C2CCC3(C(C2(CCC1O)C)C(=O)C=C4C3(CCC5(C4CC(CC5)(C)C(=O)O)C)C)C)C	Glycyrrhetinic acid was shown to inhibit 11β-hydroxysteroid dehydrogenase, thus inhibiting the conversion of cortisol.
Glycyrrhizin	CC1(C2CCC3(C(C2(CCC1OC4C(C(C(C(O4)C(=O)O)O)O)OC5C(C(C(C(O5)C(=O)O)O)O)O)C)C(=O)C=C6C3(CCC7(C6CC(CC7)(C)C(=O)O)C)C)C)C.N.N. N	Glycyrrhizin was approved for use as a flavor and aroma in manufactured foods.
Amygdalin	C1=CC=C(C=C1)C(C#N)OC2C(C(C(C(O2)COC3C(C(C(C(O3)CO)O)O)O)O)O)O	Amygdalin was reported to suppress oxidative damage.
Porphyrin	C1=CC2=CC3=CC=C(N3)C=C4C=CC(=N4)C=C5C=CC(=N5)C=C1N2	Porphyrin constitutes a part of the heme in the hemoglobin and myoglobin.
Bicalutamide	CC(CS(=O)(=O)C1=CC=C(C=C1)F)(C(=O)NC2=CC(=C(C=C2)C#N)C(F)(F)F)O	The antiandrogen drug bicalutamide is primarily used to treat prostate cancer.

**Table 5:** Pathogenicity constraints predication for the identified compounds.

Compound	Mutagenicity	Tumorgenicity	Irritant	Reproductive Effective
Acebutolol	No	No	No	No
beta-D-fructofuranose	No	No	No	Yes
Elatin (flavonoid)	Yes	Yes	No	No
Coumarin	Yes	Yes	No	Yes
resveratrol	No	No	No	Yes
Glycyrrhetinic acid	No	No	No	No
glycyrrhizin	No	No	No	No
Amygdalin	Yes	No	Yes	Yes
Porphyrin	No	No	No	No
Bicalutamide	No	No	No	No

**Table 6:** Comparison of repurposed drugs based on their physical and chemical characteristics.

Compound	Clog	Solubility	Mol Weight	Tpsa	Drug-Likeness	Drug Score	BBB	Violations
Acebutolol	1.7	−3.5	336.0	87.66	4.91	0.83	BBB-	0
beta-D-fructofuranose	−2.7	0.38	180	110	−2.56	0.32	BBB-	0
Elatin (flavonoid)	−1.91	−1.89	594	267.2	0.42	0.14	BBB-	3
Coumarin	1.5	−2.37	146	26	−1.83	0.12	BBB+	0
resveratrol	2.83	−2.86	228.0	60.69	−3.25	0.27	BBB+	0
Glycyrrhetinic acid	5.36	−5.78	470	74.6	−2.36	0.2	BBB-	1
Glycyrrhizin	0.39	−5.14	822	267.0	−4.29	0.19	BBB-	3
Amygdalin	−3.08	−1.12	457	202.3	−8.7	0.09	BBB-	2
Porphyrin	2.05	−4.34	310.0	52.54	0.97	0.67	BBB+	1
Bicalutamide	2.14	−5.08	430	115	−11	0.24	BBB+	0

**Table 7:** Repurposed components arranged based on their capabilities.

Repurposed Components	Antioxidants	Th17 Interaction	Known Effects on Depression
Acebutolol	Yes [45]	Unknown	Unknown
beta-D-fructofuranose	Unknown	Unknown	Unknown
Elatin (flavonoid)	Yes [46]	Yes [46]	Yes [47]
Coumarin	Yes [48]	Yes [49]	Yes [50]
Resveratrol	Yes [51]	Yes [52]	Yes [53]
Glycyrrhetinic acid	Yes [54]	Yes [55]	Yes [56]
Glycyrrhizin	Yes [54]	Yes [55]	Yes [56]
Amygdalin	Yes [57]	Yes [58]	unknown
Porphyrin	Yes [31]	Yes [44]	Yes [31] (enhance prognosis)
Bicalutamide	No [59]	Yes [42]	Yes (worsen prognosis)

## Data Availability

The dataset used in this manuscript is available through the following link: https://data.mendeley.com/datasets/whr7wrh42y/1.

## Supplementary Materials

The following are available online at https://www.mdpi.com/article/10.3390/molecules27196453/s1, Table S1: Relative time efficiency of the models used. Table S2: Relative memory efficiency of the models used (for the local memory). Table S3: Relative memory efficiency of the models used (on Google cloud).
