# Supplementary material for: Adera2.0: A Drug Repurposing Workflow for Neuroimmunological Investigations Using Neural Networks
Source: Molecules. 2022 Sep 30;27(19):6453. doi: 10.3390/molecules27196453 (PMC9571571; doi:10.3390/molecules27196453)
Supplement: Supplementary file 1 [file molecules-27-06453-s001.zip › molecules-1883062-supplementary.pdf]

Table S1. Relative time efficiency of the models used.

Model	AVS* TIME (ms)	Input time(ms)	Kernel launch time(ms)	Device compute time	TF placement Host/device	Time spent on eager execution (ms)
A	10.6	0.2	6.1	3.3	49	98
B	4.3	0.3	2.1	0.5	42/58	93
C	4.8	0.3	2.4	0.6	43/56	94
D	4.6	0.3	2.3	0.6	38/61	92
E	5.1	0.3	3.3	1.4	39/60	94
F	6.5	0.2	4.0	2.2	34/ 65	98
G	6.2	0.5	3.9	1.6	40/59	94
H	6.8	0.1	4.6	1.9	29/70	98

*AVS Average Step Time

Table S2. Relative memory efficiency of the models used (for the local memory)

	Allocation	Deallocation	Memory Capacity	Peak Heap Usage	Peak Memory Usage
A	452	548	13.45 GiB	1.18 GiB	1.18 GiB
B	486	514	13.45 GiB	1.11 GiB	1.11 GiB
C	476	524	13.45 GiB	1.12 GiB	1.12 GiB
D	480	520	13.45 GiB	1.13 GiB	1.13 GiB
E	488	512	13.45 GiB	1.15 GiB	1.15 GiB
F	471	529	13.45 GiB	1.15 GiB	1.15 GiB
G	464	536	13.45 GiB	1.15 GiB	1.14 GiB
H	480	520	13.45 GiB	1.18 GiB	1.17 GiB

Table S3. Relative memory efficiency of the models used (on Google cloud)

	Allocation	Deallocation	Memory Capacity	Peak Heap Usage	Peak Memory Usage
A	547	453	64 GiB	167 KiB	31 KiB
B	459	459	64 GiB	167 KiB	9 KiB
C	556	444	64 GiB	167 KiB	14.00 KiB
D	459	459	64 GiB	167 KiB	18.75 KiB
E	516	484	64 GiB	167 KiB	28.00 KiB
F	553	447	64 GiB	167 KiB	14.25 KiB
G	568	432	64 GiB	167 KiB	22.00 KiB
H	550	450	64 GiB	167 KiB	21.50 KiB
